# Pennsylvania State Dental Society

**Published:** 1886-10

**Authors:** Wm. H. Trueman


### PENNSYLVANIA STATE DENTAL SOCIETY.

EIGHTEENTH ANNUAL MEETING, HELD AT CRESSON, JULY 27, 28, AND 29, 1886.

REPORTED FOR THE INDEPENDENT PRACTITIONER, BY WM. H. TRUEMAN, D. D. S.

### WEDNESDAY MORNING SESSION.

Dr. J. C. M. Hamilton, of Tyrone, read a paper upon

### THE DENTAL PULP AND ITS TREATMENT.

He remarked that inflammation of the dental pulp does not differ materially from inflammation elsewhere, and is accompanied by similar symptoms. The first step from the normal condition of a pulp exposed by caries is, when responsive to thermal changes, or to pressure, or from other causes, there is an increased flow of blood, marked by pain of long or short duration, or spasmodic with increasing severity. In the earlier stages the pain may be general and difficult to locate, but it usually becomes more fixed as the disease advances.

In the earlier stages of the disease, after locating the seat of trouble, the application within the cavity of carbolic acid, oil of cloves, opium in its various forms, menthol, chloroform, etc., will often give relief. A preparation of pure wood creosote and oil of cloves, of each half an ounce, adding from ten to twenty grains of acetate of morphia, he recommended in these cases, having used it for a long time with most satisfactory results. He referred to an article in the June number of the *Dental Cosmos*, in which the writer condemns pulp capping as inapplicable to a pulp that has once been inflamed, even in its early stages, and advises devitalization in all such cases. Of this he did not approve. While in the later stages of the disease the risk is so great that it may be better to devitalize at once, he condemns most heartily the wholesale destruction of pulps there advised. In all cases of pulp exposure where no previous irritation has existed, or when the inflammation has not advanced beyond the first stages, he carefully prepares the cavity and in simple cases caps at once, using for this purpose a cement. If, however, inflammation is present, he allows a small pledget of cotton saturated with the preparation just given to remain in the cavity twenty-four hours at least, securely covered with cotton and sandarach varnish, after which the capping may be applied. Oxide of zinc, made into a paste with the creosote and morphia preparation, he has used as a pulp capping for the last two years, with very satisfactory results, carefully covering it with a cement, which in turn is covered with a more durable material after the lapse of a few weeks or months.

The same preparation, with arsenic added, he uses to devitalize pulps, securing by its use entire freedom from the excruciating pain so frequently following the use of arsenic alone.

Dr. H. C. Register, of Philadelphia, read a paper upon

COMPRESSED AND WARM AIR AS A GERMICIDE, PAIN OBTUNDER, AND OTHERWISE USEFUL AGENT IN DENTAL PRACTICE.

He claimed no originality in the use of warm air, or air under pressure, in dental practice. That honor belongs to the inventor of the air syringe in general use. Several professional friends, whom he named, had used compressed air in much the same way that he would presently describe, but only to a limited extent. He had been assured by them that he had carried his investigations of the air system much farther than they had, and finding daily good results from its use, he felt it a duty to make it known. While there is yet a diversity of opinion, it is generally accepted by the profession that micro-germs have much to do with the production of caries; this has been so clearly shown by the investigations of Dr. Miller, of Berlin, that Prof. Black, referring to them, says: "It was the most perfect work of the kind that the world ever saw. His experiments were so scientifically conducted, and the products so carefully analyzed, that he left not a hook upon which a tenable objection could be hung." With these investigations, we, as a profession, have much to do. If, by reducing these invaders of the oral territory to the minimum, we get rid of the most persistent cause of caries of the teeth, it is, therefore, meet and right that a sanitary system should be introduced into every household, to that end. Flushing the oral cavity at least once a day, with an alkaline wash, by atomization, under sufficient atmospheric pressure, would so reduce the prevalence of these destructive germs and counteract the acid found in the cavities of the teeth, that the reacting force of life would check the carious growth. He described at length the accepted theory of the origin, life history, and relation of micro-germs to dental caries, showing that moisture of a ferment character was essential to their existence, and contended that those germs which could not be washed away by atomization would be destroyed by a continuous current of air heated to 120 degrees. He suggested using this warm air current immediately before filling, in order not only to destroy any germs that may be beyond the walls of the cavity, but also to render this portion of the tooth less favorable to their existence, and to secure a more perfect adaptation of the filling.

He found the continuous warm air current an excellent pain obtunder, in sensitive dentine. In preparing for filling he adopted the following method: After opening the cavity, using the atomizer under strong pressure, he carefully washed the cavity and surrounding parts with listerine, or other like liquid. The dam is now applied and the cavity slightly dried with warm air. At first this is irritating, and sometimes produces a slight pain during its application. The thin walls are now broken down and the decay removed. If the tooth continues sensitive, it is bathed in equal parts of carbolic and tannic acid, atropia, or other like obtunder. The warm air, at about blood heat, is now applied, very gently at first, and stronger as the patient can bear it. In a few minutes sensation will be so far reduced that considerable cutting may be done, producing in most cases no pain at all. After the cavity is shaped, the air is applied and thorough dryness secured. While in this condition he is in the habit of covering the dentine, in large cavities, with a film of gutta-percha or collodion. In deep cavities phosphate of zinc may be used at the bottom and thoroughly air-dried. Cold air, which previously could not be tolerated, can now be thrown into the cavity without producing the slightest irritation. In small cavities this care is not necessary.

Devitalized teeth, to which there is no fistulous opening, but which contain a putrescent pulp, should be opened with as little pressure upon its contents as possible, making a large and free opening into the bulbous portion of the pulp cavity. The atomizer should now be brought into use under low pressure, and the putrescent matter washed out, using for this purpose any preferred wash. The dam is now applied, and air heated to the degree of toleration gently forced into the whole extent of the canals. After dryness has been accomplished, a nerve broach may be employed to carefully enter the canals and detach the dried portions, the air being allowed to fill the canals at the same time, and to lift or blow these portions out. After this has been done, he uses a mixture of iodide of zinc and permanganate of potash, of a creamy consistency, taking a small portion upon a broach, and by a gentle rotary motion forcing it into the canals, after which the warm air is applied to drive it yet further. If there now remains the slightest odor of decomposition, the treatment is repeated, or a drop or two of aromatic sulphuric acid is flowed into the canals and the warm air again force in under low pressure. The canals are now filled with oxychloride of zinc, to the oxide of which is added a few grains of iodoform. In treating putrescent pulps with a fistulous track, the same care in cleaning the canals is not required. The atomizer may be applied under strong pressure to wash out the track with tepid water, after which the desired escharotic may be used, it also being forced through by the same means. Aromatic sulphuric acid he accepted as the best remedy for general use. He objected to the use of carbolic acid at this stage, as it is not a solvent. Its after use may, however, be indicated. An over-sanguine faith in carbolic acid has, he believed, caused about as much trouble in dental practice as it has done good.

He employs the atomizer in treating pyorrhœa, using heavy air pressure to force the remedy well into the pockets, and advises its constant use, after treatment had ceased, to keep the spaces between the teeth clean and in a healthy condition. His method is as follows: First, cleanse the necks of the teeth from all foreign substances, using a germicide with the atomizer, throwing it deep into the pockets, so as to wash out all loose matter. The air is now used with a simple blow-pipe; the gums being held back by the force of the air jet, the parts are fully open to view while all hard matter is cut or scraped away down to the healthy peridental membrane by means of scalers. If the character of the detached gum calls for it, amputate those portions which are known to have lost all possible attachment to the teeth, or cut them away in such manner as to promote granulations. He recommends that the patient be provided with an apparatus, so as to keep up the treatment of atomization after each meal, and on retiring.

He uses the warm air with marked advantage in treating discolored teeth. After dressing the root and closing the opening at the apex, while the rubber dam is in position, he first relieves the tooth of as much defective tissue as the case calls for, when the warm air is thrown into every part under a pressure of from twenty to twenty-five pounds to the square inch, being heated to as high a temperature as the patient can bear. After from two to five minutes drying he makes an application of aromatic sulphuric acid of full strength, allowing it to remain a minute or two. He desires to get the solvent action of the acid; any other agent that will act upon the discolored matter and open the tubuli, and yet not act too quickly upon the dentine, would probably answer as well. The warm air is again applied, and the tooth structure rendered as dry as possible. After two or more applications of the air and acid alternately, the cavity is washed out with the atomizer under heavy pressure, catching the liquid in a napkin, so as to avoid disturbing the rubber dam. The tooth is now carefully dried, and the cavity saturated with Labarraque's solution of chloride of lime. This preparation acts both as a bleaching agent and as an alkali to neutralize any acid that may remain. The warm air is again applied until the tooth is perfectly dry; the solution of lime is reapplied, and the tooth again dried, this being repeated several times. Any bleaching agent may be used. The advantage of the air drying system consists in getting rid of the moisture in the distal portions of the tooth, so that the solvent and bleaching agents may follow up the discolored matter. The manipulations here described may probably take about half an hour. When the color is satisfactory, the cavity, in greater part, is filled with gutta-percha, or zinc phosphate cement, and finished with gold filling. Cases treated in this way retain their restored color, and look well.

In conclusion, he earnestly recommended the use of an atomizer in addition to the tooth brush for daily use, a harmless alkali like lime water, or a mild germicide mouth wash being used under a pressure of from ten to thirty pounds; it is quickly done, and under that pressure the liquid is broken up and forced between the teeth, not only removing any remains of food, etc., but most effectively neutralizing any destructive agent. The doctor illustrated its use for this purpose. The effect is rather pleasant, and with a suitable mouth wash leaves a sensation of cleanliness that is very gratifying, being much more pleasant than that felt after a thorough scrubbing with a tooth brush.

The air-compressing apparatus used by the doctor resembles closely the well-known Burgess Mechanical Blowpipe, and consists of an air pump, worked by a pedal, over which is mounted the air reservoir, provided with a pressure-gauge and suitably arranged openings, to which a flexible rubber tube may be secured, the pressure being controlled by a stop-cock. Several sizes are upon the market, and being intended for office use they are handsomely gotten up. The doctor exhibited quite a collection of atomizing tubes of various shapes, and also a number of blow pipes designed for special uses. The most of them were experimental. He finds that but few are really needed. They are attached to the air compressor by a flexible rubber tube, and each is provided with a stop-cock readily controlled by the fingers of the hand that holds it, so that the air current is always under control. The apparatus is not expensive, quite simple and easily managed, and if as useful as it promises to be, is well worth having.

The reading of the paper was followed by an animated discussion, more, however, in the nature of questions concerning the use of the various pieces of apparatus exhibited, and their special uses, than upon the system itself. That was so novel, and having been used by no one present except the essayist, excited considerable interest.

The election of officers was now held, with the following results:

*President*—E. P. Kremer, Lebanon.

*First Vice-President*—H. C. Register, Philadelphia.

*Second Vice-President*—W. F. Fundenberg, Pittsburg.

*Recording Secretary*—Wm. B. Miller, Altoona.

*Assistant Secretary*—Jos. R. C. Ward, Philadelphia.

*Corresponding Secretary*—W. H. Fundenberg, Pittsburg.

*Treasurer*—J. C. M. Hamilton, Tyrone.

*Board of Censors*—J. S. Goshorn, W. E. Van Orsdel, G. L. Robb, C. S. Beck, A. Boice.

*Board of Examiners*—J. C. Green, and W. E. Magill, re-elected.

Next place of meeting, Glen Summit, Luzerne Co., with Pittsburg as an alternate, if suitable arrangements at Glen Summit cannot be made.

(TO BE CONTINUED.)

